# Light-induced silencing of neural activity in Rosa26 knock-in and BAC transgenic mice conditionally expressing the microbial halorhodopsin eNpHR3

**DOI:** 10.1038/s41598-020-59984-3

**Published:** 2020-02-21

**Authors:** Itaru Imayoshi, Sawako Tabuchi, Mami Matsumoto, Satsuki Kitano, Hitoshi Miyachi, Ryoichiro Kageyama, Akihiro Yamanaka

**Affiliations:** 1 Research Center for Dynamic Living Systems, Graduate School of Biostudies, Kyoto University, Kyoto, 606-8501 Japan; 2 Institute for Frontier Life and Medical Sciences, Kyoto University, Kyoto, 606-8507 Japan; 3 World Premier International Research Initiative–Institute for Integrated Cell-Material Sciences, Kyoto University, Kyoto, 606-8501 Japan; 4 Research Institute of Environmental Medicine, Nagoya University, Nagoya, 464-8601 Japan

**Keywords:** Expression systems, Molecular neuroscience

## Abstract

An engineered light-inducible chloride pump, *Natronomonas pharaonis* halorhodopsin 3 (eNpHR3) enables temporally and spatially precise inhibition of genetically defined cell populations in the intact nervous tissues. In this report, we show the generation of new mouse strains that express eNpHR3-EYFP fusion proteins after Cre- and/or Flp-mediated recombination to optically inhibit neuronal activity. In these mouse strains, Cre/Flp recombination induced high levels of opsin expression. We confirmed their light-induced activities by brain slice whole-cell patch clamp experiments. eNpHR3-expressing neurons were optically hyperpolarized and silenced from firing action potentials. In prolonged silencing of action potentials, eNpHR3 was superior to eNpHR2, a former version of the engineered pump. Thus, these eNpHR3 mouse strains offer reliable genetic tools for light-induced inhibiting of neuronal activity in defined sets of neurons.

## Introduction

Optogenetic technics for artificially regulating neuronal activities by light are widely employed for manipulating the nervous system^1–3^. Optogenetic strategies use light to artificially regulate cellular functions in targeted cells *in vivo* with high temporal and spatial precision^4–7^. Most of the optogenetic tools in the field of neuroscience are based on light-activatable transporters or ion channels. Ligh-triggered activation of microbial opsins depolarize/hyperpolarize neuronal membranes, then inducing temporally precise activation/inhibition of targeted neurons^1–3,8^.

Light-controllable inhibition of neuronal activities can be accomplished by expressing the halorhodopsin derived from *Natronomonas pharaonis* (NpHR), a fast, light-sensitive chloride pump. In response to yellow light, NpHR inhibits neuronal activity via chloride influx^9–12^. The original NpHR has undergone molecular modifications to improve its surface membrane localization and reduce intracellular aggregation by attaching endoplasmic reticulum export and neurite trafficking motifs^11,12^. These second- and third-generation enhanced tools (eNpHR2 and eNpHR3) have been successfully applied *in vivo* and in nervous system in a number of studies^13–18^. Here, we generated and characterized new knock-in/BAC transgenic mouse strains that express eNpHR3-EYFP fusion proteins after Cre- and/or Flp-mediated recombination to optically inhibit neuronal activity. We also compared the eNpHR3-knock-in mice to the previously generated eNpHR2-knock-in mice^19^ under the same experimental conditions, and confirmed the superior ability of the eNpHR3-knock-in/BAC transgenic mice in light-triggered silencing of neuronal activities.

## Results

**Figure 1:**
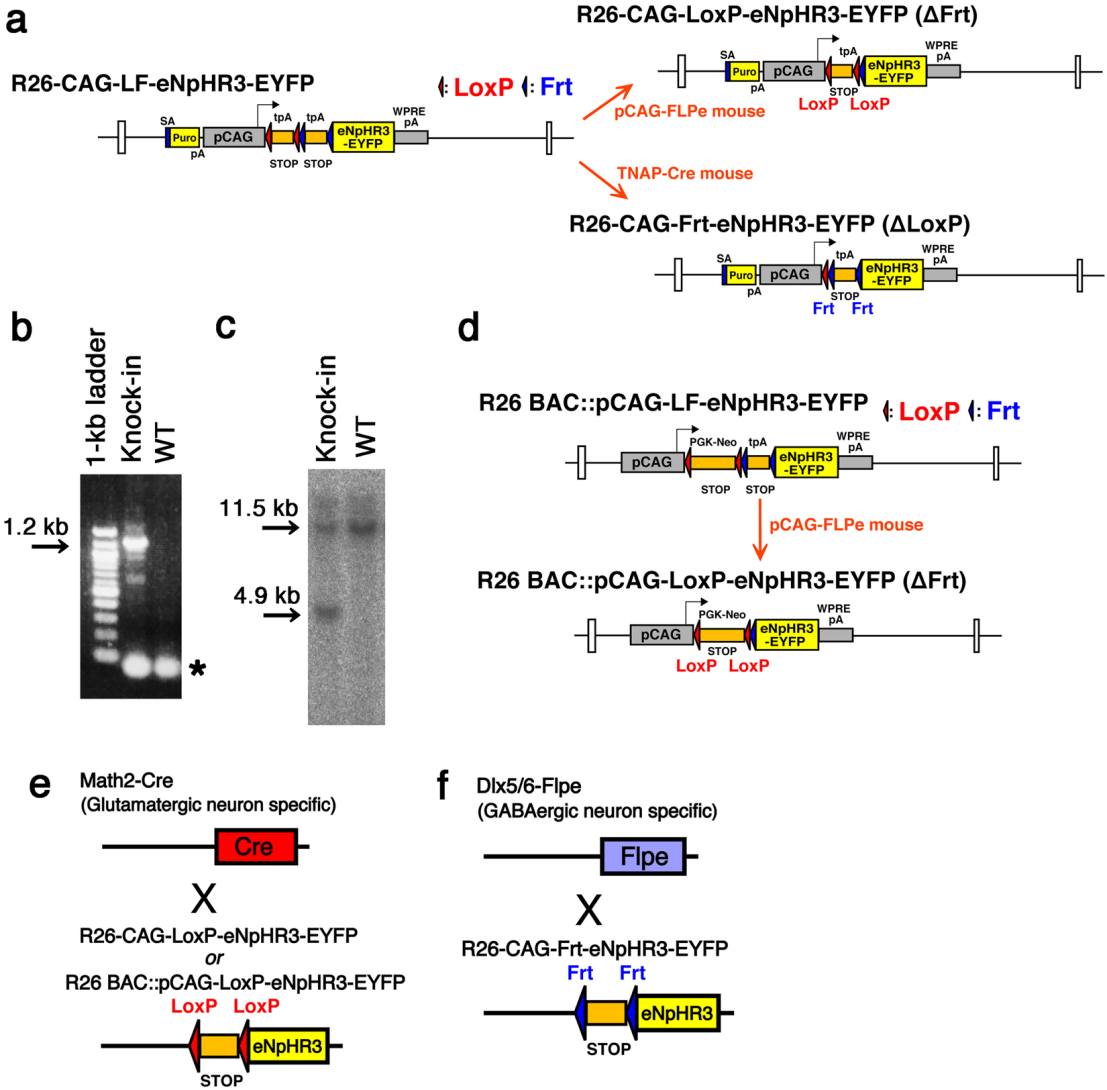
Schematic illustration of Cre- and/or Flp-recombinase-dependent eNpHR3-EYFP expression. (**a**) The transgene cassette is composed of a splice acceptor sequence (SA)-puromycin resistance gene (yellow), the CAG promoter, a floxed (red triangles) 3x SV40pA stop cassette, a 3x pA (SV40pA-TKpA-SV40pA) stop cassette flanked by Frt sites (blue triangles), eNpHR3-EYFP and WPRE-SV40pA were knocked in at the Rosa26 locus (R26-CAG-LF-eNpHR3-EYFP). R26-CAG-LF-eNpHR3-EYFP was modified by germline excision of the Frt-flanked stop cassette by pCAG-FLPe mouse to produce a Cre-dependent eNpHR3-EYFP mouse strain (R26-CAG-LoxP-eNpHR3-EYFP). Germline excision of the floxed stop cassette by TNAP-Cre mouse converts R26-CAG-LF-eNpHR3-EYFP to a Flp-dependent eNpHR3-EYFP mouse strain (R26-CAG-Frt-eNpHR3-EYFP). (**b**,**c**) Representative results of PCR (**b**) and Southern blot hybridization analysis (**c**) of the genomic DNA from embryonic stem cells, in which successful homologous recombination at the Rosa26 locus was induced. The PCR product of 1.2 kb was amplified from the R26-CAG-LF-eNpHR3-EYFP knock-in allele (**b**). An asterisk mark indicates the primed dimers. The genomic DNA derived from the embryonic stem cell clones for the Southern blot was digested with *EcoRV* to reveal the wild-type (WT) 11.5-kb allele and the R26-CAG-LF-eNpHR3-EYFP knock-in allele of 4.9 kb (**c**). The whole images of PCR and Southern blot hybridization analysis were shown in Fig. S1. (**d**) Schematic illustration of the R26 BAC::pCAG-LF-eNpHR3-EYFP transgenic strain and its derivative. Similar to the eNpHR3-knock-in strain, the Cre-dependent R26 BAC::pCAG-LoxP-eNpHR3-EYFP strain was obtained by germline deletion of the floxed transcription cassette. In the eNpHR3-BAC transgenic strains, the floxed transcription cassette also has a PGK-Neo expression sequence. This elongated the DNA sequences between the two loxP sites from 791 bp in the eNpHR3-knock-in strains to 2,521 bp, and possibly changed the sensitivity to Cre-mediated recombinations. **(e,f**) Schematic illustrations of glutamatergic and GABAergic neuron-specific eNpHR3 expressions by the Math2-Cre knock-in (**e**) and Dlx5/6-Flpe transgenic strains (**f**), respectively.

We developed dual recombinase-responsive knock-in mouse strains, in which eNpHR3-EYFP is conditionally expressed from the Rosa26 locus after Cre- and Flp-mediated recombination (R26-CAG-LF-eNpHR3-EYFP, Fig. 1a–c). The main structure of the targeting vector is composed of a ubiquitous CAG promoter, LoxP-flanked primary and Frt-flanked secondary stop cassettes, and eNpHR3-EYFP. In mouse embryonic stem cells, the transgene construct was inserted by homologous recombination into the Rosa26 locus, which is reportedly transcriptionally active in almost all cells of the body at all developmental, postnatal and adult stages^20^. This was previously confirmed by mTFP1 fluorescent protein expression with the same Rosa26 knock-in strategy^21^. To induce high-level transgene expression, we applied the CAG promoter (a combination of the chicken beta-actin promoter and the cytomegalovirus immediate-early enhancer). A woodchuck hepatitis virus posttranscriptional regulatory element (WPRE) sequence was also integrated into the 3′UTR region to enhance transgene expression levels by mRNA stabilization^17^. By germline removal of the Frt-flanked stop cassette in R26-CAG-LF-eNpHR3-EYFP, a Cre-dependent eNpHR3-EYFP line (R26-CAG-LoxP-eNpHR3-EYFP) was generated. Similarly, R26-CAG-LF-eNpHR3-EYFP was modified by germline removal of the LoxP-flanked stop cassette to generate an Flp-dependent eNpHR3-EYFP line (R26-CAG-Frt-eNpHR3-EYFP).

**Figure 2:**
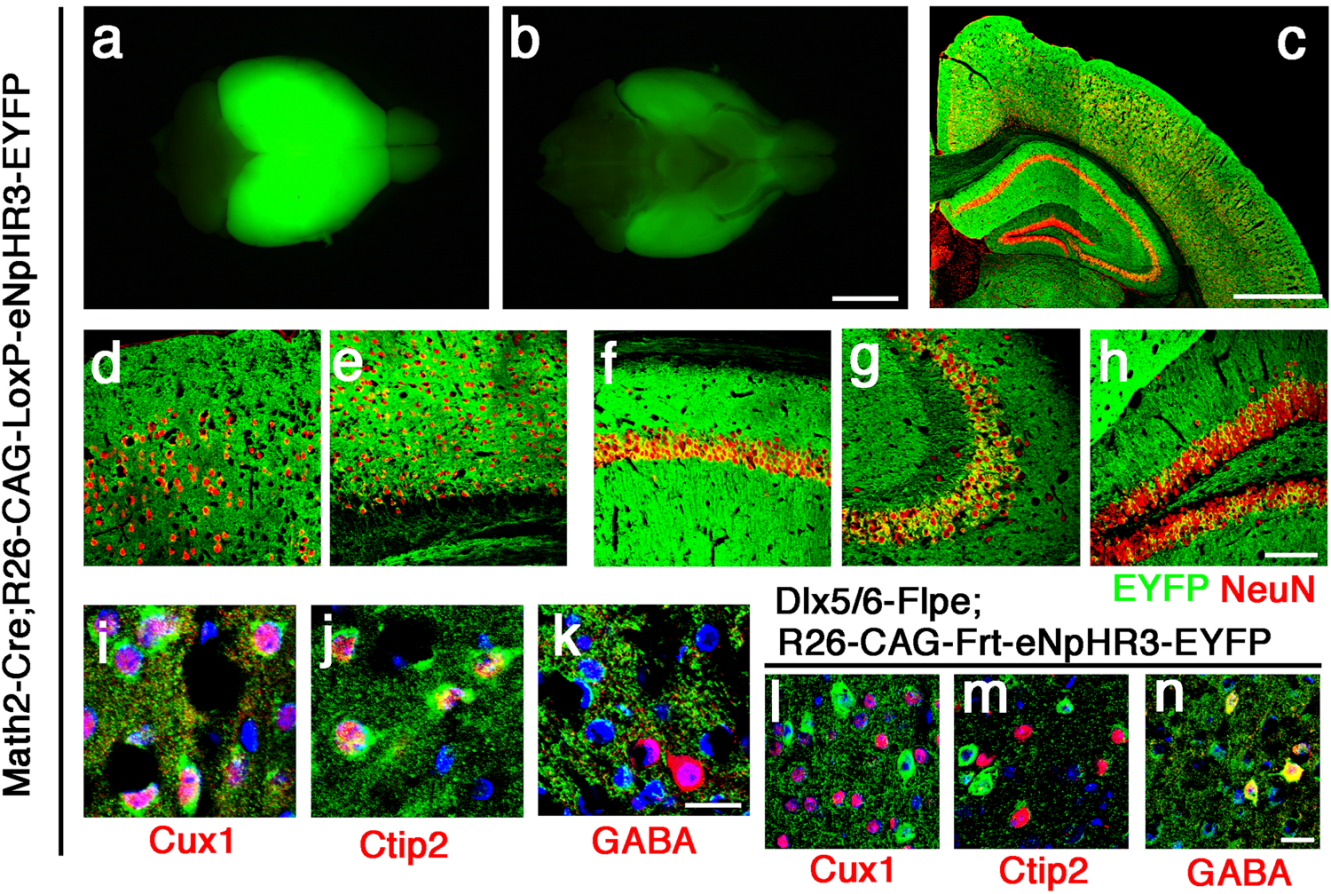
Conditional eNpHR3-EYFP expression in the forebrain glutamatergic and GABAergic neurons. (**a**–**k**) R26-CAG-LoxP-eNpHR3-EYFP mice were crossed with Math2/Nex-Cre mice. Dorsal (**a**) and ventral (**b**) view of the whole brain from the adult double transgenic mice, indicating strong and broad expression of the eNpHR3-EYFP fusion protein. (**c–h**) Coronal sections of the neocortex and hippocampus regions (**c**) were immunostained with anti-GFP/EYFP (*green*), anti-NeuN (*red*) antibodies. The upper (**d**) and deep (**e**) layers of the neocortex, and CA1 (**f**), CA3 (**g**), and dentate gyrus (**h**) regions of the hippocampus are enlarged. (**l**–**n**) R26-CAG-FRT-eNpHR3-EYFP mice were crossed with Dlx5/6-Flpe mice. (**i**–**n**) Immunostaining of the neocortex with anti-GFP/EYFP (*green*), anti-Cux1 (**i,l**), anti-Ctip2 (**j,m**), and anti-GABA (**k,n**) antibodies (*red*). Scale bars represent 3 mm in (**a**,**b**), 600 μm in (**c**), 100 μm in (**d**–**h**) and 20 μm in (**i**–**n**).

To check the conditionally induced expressions of eNpHR3-EYFP in the brain, we first crossed Cre-dependent R26-CAG-LoxP-eNpHR3-EYFP mice with Math2/Nex-Cre knock-in driver strain, that induces efficient recombination specifically in forebrain glutamatergic/excitatory neurons^19,21,22^ (Fig. 1a,e, Fig. 2). The specific recombination in glutamatergic neurons by the Math2-Cre driver strain was previously confirmed by eNpHR2-EYFP expression with the same Rosa26 knock-in strategy^19^. In Math2-Cre;R26-CAG-LoxP-eNpHR3-EYFP double transgenic mice, strong naive EYFP fluorescence was detected throughout the adult brains (Fig. 2a,b). To analyze expression patterns of the eNpHR3-EYFP fusion product in more detail, coronal brain sections of the adult double transgenic mice were stained with the antibody against EYFP. eNpHR3-EYFP expression was detected in NeuN-expressing neurons both in the upper and deep layers (Fig. 2c,d,e) of the neocortex. eNpHR3-EYFP expression was also broadly detected in projection neurons in the hippocampus (Fig. 2c,f,g,h). We also confirmed eNpHR3-EYFP expressions in upper layer-marker Cux1 + neurons and deep layer-marker Ctip2 + neurons (Fig. 2i,j), but not in GABA + inhibitory neurons (Fig. 2k).

We also crossed Flp-dependent R26-CAG-Frt-eNpHR3-EYFP mice with Dlx5/6-Flpe driver transgenic strain, that induces specific recombination restrictedly in forebrain inhibitory GABAergic interneurons^23^ (Fig. 1f). We observed specific eNpHR3-EYFP expressions in GABA-immunopositive neurons throughout the neocortex (Fig. 2n), but not in glutamatergic neurons (Fig. 2l,m), as reported previously^19^. We then validated the effects of yellow-light illumination on neural activities in the neocortical glutamatergic and GABAergic neurons expressing eNpHR3.

**Figure 3:**
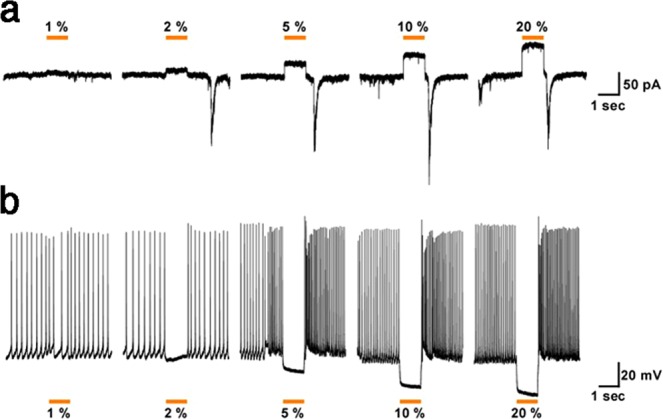
Light-induced silencing of glutamatergic neurons in brain slices from R26-CAG-LoxP-eNpHR3-EYFP mice. Yellow light inhibited the activity of eNpHR3-expressing neocortical glutamatergic neurons. (**a**) Under whole-cell voltage-clamp mode, yellow light induced outward photocurrents in a light intensity-dependent manner (at 1, 2, 5, 10, and 20%; from left to right). (**b**) Yellow light illumination completely inhibited spontaneous action potentials in eNpHR3-expressing neurons (representative traces in current-clamp mode) in a light intensity-dependent manner (at 1, 2, 5, 10, and 20%; from left to right).

To determine the functional effects of eNpHR3-EYFP expressed by glutamatergic neurons in the neocortex of Math2-Cre;R26-CAG-LoxP-eNpHR3-EYFP double transgenic mice, slice patch clamp analyses were conducted. We analyzed eNpHR3-EYFP-expressing glutamatergic neurons randomly selected from the upper and deep layers of the neocortex. Fluorescent eNpHR3-EYFP-expressing neurons were whole-cell patch clamped and yellow light was irradiated through the objective lens of the microscope. Various intensities of yellow light illumination rapidly induced outward currents (Fig. 3a) and completely silenced the generation of spontaneous action potentials (Fig. 3b) in a light dose dependent manner.

**Figure 4:**
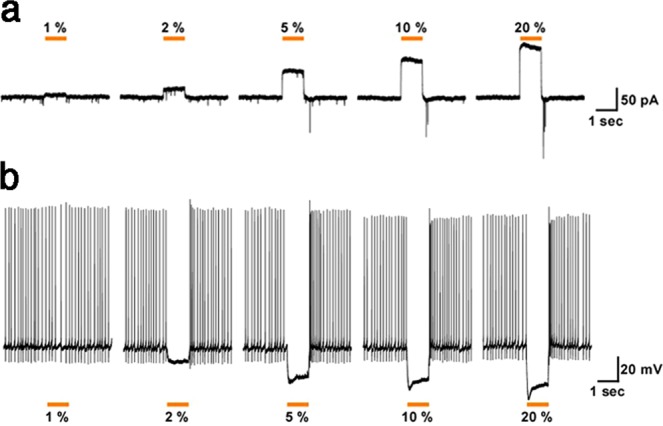
Light-induced silencing of GABAergic neurons in brain slices from R26-CAG-Frt-eNpHR3-EYFP mice. Yellow light inhibited the activity of eNpHR3-expressing neocortical GABAergic neurons. (**a**) Under whole-cell voltage-clamp mode, yellow light induced outward photocurrents in a light intensity-dependent manner (at 1, 2, 5, 10, and 20%; from left to right). (**b**) Yellow light illumination completely inhibited spontaneous action potentials in eNpHR3-expressing neurons (representative traces in current-clamp mode) in a light intensity-dependent manner (at 1, 2, 5, 10, and 50%; from left to right).

To determine whether conditionally expressed eNpHR3-EYFP is also effective in GABAergic interneurons, we conducted patch clamp analyses in acute brain slices of the neocortex from Dlx5/6-Flpe;R26-CAG-Frt-eNpHR3-EYFP double transgenic mice. We randomly analyzed eNpHR3-EYFP-expressing GABAergic neurons throughout the neocortex. Similar to the results in glutamatergic neurons, yellow light exposure rapidly induced outward currents (Fig. 4a) and completely silenced the generation of spontaneous action potentials in GABAergic neurons (Fig. 4b) in a yellow light dose-dependent manner.

**Figure 5:**
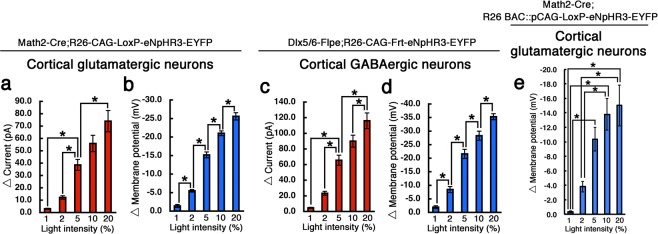
Summary of eNpHR3-EYFP functional properties in neocortical glutamatergic and GABAergic neurons. (**a**,**b**) Bar graphs summarize yellow light-induced outward currents (**a**) and hyperpolarization (**b**) in eNpHR3-expressing glutamatergic neurons of the Math2-Cre;R26-CAG-LoxP-eNpHR2-EYFP double transgenic mice. (**c**,**d**) Bar graphs summarizing yellow light-induced outward currents (**c**) and hyperpolarization (**d**) in eNpHR3-expressing GABAergic neurons of the Dlx5/6-Flpe;R26-CAG-Frt-eNpHR3-EYFP double transgenic mice. (**e**) Bar graphs summarizing yellow light-induced outward currents in eNpHR3-expressing glutamatergic neurons of the Math2-Cre;R26 BAC::pR26-CAG-LoxP-eNpHR3-EYFP double transgenic mice. Data represent means of 12 (**a**), 15 (**b**), 12 (**c**), 10 (**d**), and 7 (**e**) neurons; error bars represent standard error (SE). *p < 0.05, one-way ANOVA with Tukey’s test.

A summary of eNpHR3-EYFP functional properties in the knock-in mice determined by whole-cell patch clamp analyses in neocortical glutamatergic and GABAergic neurons is displayed in Fig. 5. The magnitude of eNpHR3-mediated hyperpolarization and photocurrent correlated with the light intensity (Fig. 5a–d). The functional effects of eNpHR3 in glutamatergic/pyramidal neurons and GABAergic interneurons were largely similar, suggesting uniform Cre/Flp-dependent eNpHR3-EYFP expression levels in both lines of knock-in mice.

Ubiquitous and uniform transgene expressions in the Rosa26 locus were reportedly observed in the case of bacterial artificial chromosome (BAC) transgenic mice, in which EGFP was expressed under the regulation of a 187-kb BAC containing the mouse Rosa26 locus^24^. To determine whether this Rosa26 BAC functions as a platform of conditional transgene expression cassette, we also generated R26 BAC::pCAG-LF-eNpHR3-EYFP and R26 BAC::pCAG-LoxP-eNpHR3-EYFP transgenic mice (Fig. 1d), and confirmed the eNpHR3-EYFP functions in the brain slices. Similar to the eNpHR3-EYFP knock-in mice, we observed strong expressions of eNpHR3-EYFP in glutamatergic neurons in Math2-Cre;R26 BAC::pCAG-LoxP-eNpHR3-EYFP double transgenic mice (*data not shown*). We also observed the yellow light intensity-dependent outward photocurrents in the eNpHR3-EYFP-expressing glutamatergic neurons of the BAC transgenic mice (Fig. 5e).

**Figure 6:**
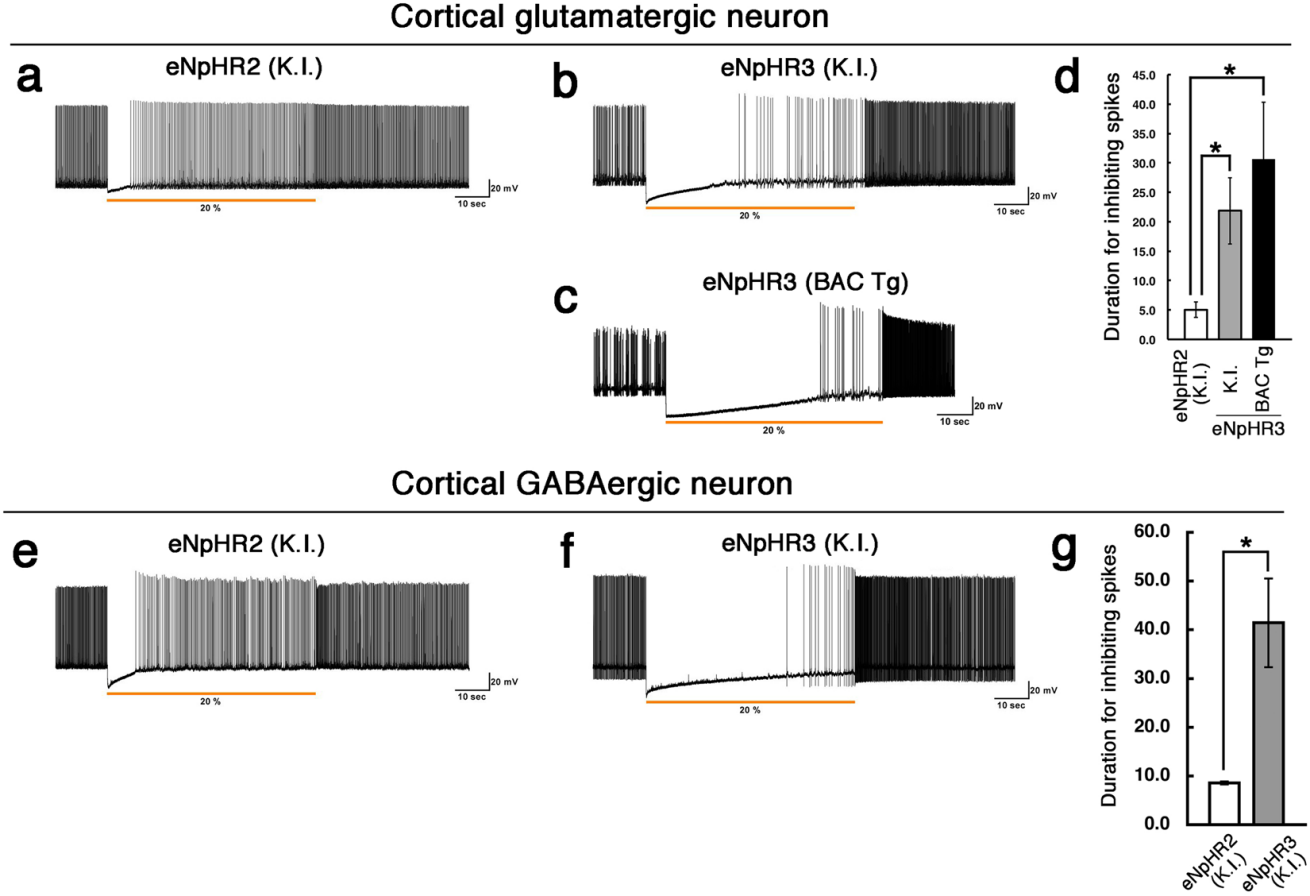
Prolonged light-induced inhibition of neuronal actives in the eNpHR3-EYFP knock-in (K.I.) and BAC transgenic (Tg) mice. (**a**–**c**) Yellow light was continuously illuminated for 1 min to glutamatergic neurons in eNpHR2-knock in mice (**a**), eNpHR3-knock-in mice (**b**), and eNpHR3-BAC transgenic mice (**c**). (**d**) The latency to recovery of spontaneous neural activities from light-induced inhibition. The duration of completely inhibiting spikes was significantly longer both in the NpHR3-knock-in and BAC transgenic mice, compared with the eNpHR2-knock-in mice. (**e**,**f**) Yellow light was continuously illuminated for 1 min to GABAergic neurons in brain slices from eNpHR2-knock-in (**e**), and eNpHR3-knock-in mice (**f**). (**g**) Similar to the case in the glutamatergic neurons, the duration of completely inhibiting spikes in GABAergic neurons was significantly longer in the NpHR3-knock-in mice, compared with the eNpHR2-knock-in mice. The data represent mean ± SE. *p < 0.05, two-tailed Student’s *t* test.

Finally, we compared long-term silencing of neural activities under prolonged yellow light-illuminations in the eNpHR3-knock-in/BAC-transgenic mice (this study) and in the previously generated eNpHR2-knock-in mice^19^. Compared to the eNpHR2-knock-in mice, the duration of completely inhibited spiking was approximately 4-fold longer in glutamatergic neurons of both the NpHR3-knock-in and BAC transgenic mice (Fig. 6a–d). Similarly, in the GABAergic neurons, the duration of completely inhibited spiking was approximately 4-fold longer in the NpHR3-knock-in mice compared with the previous NpHR2-knock-in mice (Fig. 6e–g).

## Discussion

The Rosa26 locus has been widely used when aiming for the ubiquitous expressions of the genetically encoded reporter and actuator tools in various tissues and organs^25,26^. A series of Rosa26 knock-in mice inducibly expressing optogenetic tools, including NpHRs, have been developed^17,19^. In addition to the Cre-dependent strains, dual recombinase-sensitive strains for Cre/Flp and Cre/Dre were developed for the more selective expression of optogenetic tools in restricted sets of neurons. A combination of the Cre/LoxP and Tet gene expression systems was also very successfully applied to the targeted expression of optogenetic tools. Other genetic loci, such as *ActbB* and TIGRE haves been identified as more suitable sites for TetO/TRE sequence integration than the Rosa26 locus^8,27,28^. These genetic loci are reportedly more tolerant to chromatin silencing.

Therefore, we have multiple choices of inducible mouse lines even for similar optogenetic tools. The strength of promoter activity and stability of the mRNA sequence directly affect transgene expression levels^29,30^. The sort of fluorescent protein- or epitope-tags used to identify localization of the expressed optogenetic tools may also influence the selection of mouse lines. Sometimes, it is necessary to consider differences in the easiness of the occurrence of the Cre/Flp-recombinations^31^. Differences in the lengths of the floxed transcription stop cassettes also potentially change the patterns of Cre-mediated recombination. A shorter floxed transcription stop cassette is more easily recombined than a longer stop one for the same level of Cre expression. This issue becomes more apparent when temporally inducible Cre, such as tamoxifen-inducible CreER, is used. For instance, in this study, two Cre-sensitive eNpHR3-strains were developed; the eNpHR3-knock-in strains have shorter, 791-bp stop cassettes, while the eNpHR3-BAC transgenic strains have longer, 2,521-bp floxed transcription stop cassettes. Therefore, broader and more efficient recombinations are expected in the eNpHR3-knock-in strains than in eNpHR3-BAC transgenic strain. However, undesired leaky recombination might be induced in the eNpHR3-knock-in strains even by very weakly Cre-functioning cell populations, and this may lead to a decrease in the recombination specificity. In both of the eNpHR3-knock-in and eNpHR3-BAC transgenic strains, we did not observe any leaky expression of eNpHR3-EYFP even by the immunohistochemical analysis with the anti-EYFP antibody. We also did not detect any abnormal behaviors or brain structures in the both knock-in/BAC transgenic mice, in which the expression of eNpHR3-EYFP was broadly induced. However, high-levels of the transgene expression may change the condition or healthy state of the targeted neurons, and these concerns should be carefully analyzed before applying the knock-in/BAC transgenic mouse models to the specific applications.

In conclusion, we developed two new knock-in/transgenic mouse strains that conditionally express an eNpHR3-EYFP fusion protein via Cre- and/or Flp-mediated recombination. These eNpHR3-EYFP knock-in/transgenic mouse strains offer powerful tools for yellow light-induced inhibition of neuronal activity in genetically defined sets of neurons.

## Methods

### Knock-in targeting vector construction

The R26-CAG-LF-eNpHR3-EYFP knock-in vector (Fig. 1a) contains the splice acceptor sequence (SA)-puromycin resistance gene, a CAG promoter derived from pCAGGS^32^, a floxed primary stop cassette containing three polyadenylation (pA) sequences from SV40pA, an Frt-flanked stop cassette containing three pA sequences (SV40pA-TKpA-SV40pA), and eNpHR3-EYFP-WPRE-SV40pA. The eNpHR3-EYFP sequence was derived from the pLenti-hSyn-eNpHR3.0-EYFP plasmid^12^. To generate the final targeting vector, the above transgene construct was introduced into the *PacI*/*AscI* site of the pRosa26PAS plasmid^33,34^. The resulting vector contains 5′ and 3′ homology arms of 1.1 kbp and 4.1 kbp, respectively, which target the construct to the *XbaI* site of intron 1 at the Rosa26 locus. The detailed cloning strategy and complete sequence of plasmids are available on request.

### Generation of R26-CAG-LF-eNpHR3-EYFP, R26-CAG-LoxP-eNpHR3-EYFP, and R26-CAG-Frt-eNpHR3-EYFP knock-in mice

All animal protocols were approved by the Animal Care Committee of the Kyoto University (permit numbers: Med Kyo 16216 and Lif-K18018) and were performed in accordance with the principles outlined in the Kyoto University Guide for the Care and Use of Laboratory Animals. The Rosa26-targeting strategy was similar to that utilized to generate R26-CAG-LF-mTFP1 mice^21^. In short, the targeting vector was linearized and electroporated into Tc-1 embryonic stem cells, and puromycin resistant clones were selected. Genomic DNA from drug-resistant cells was screened by PCR for homologous recombination at the Rosa26 locus, using the primers Rosa26-5armFlanking (5′- CCTAAAGAAGAGGCTGTGCTTTGG -3′) and Rosa26-SA (5′- CATCAAGGAAACCCTGGACTACTG -3′), which amplified a 1.2-kbp product. Southern blot hybridization on *EcoRV*-digested genomic DNA was used to confirm homologous recombination at the 5′ end by using a 0.14-kbp probe located outside of the 5′ homology arm^21^. The probe was generated by *EcoRI* and *HindIII* digestion of the pRosa26 5′probe plasmid. The targeted and wild-type alleles produced products of 11.5-kb and 4.9-kb, respectively. Chimeric mice were produced from two successfully targeted ES cell clones by blastocyst injection with C57BL/6 J embryos. Germ line transmission of the targeted allele was assessed by EYFP PCR with primers (5′- GTAAACGGCCACAAGTTCAGCGTGTC -3′) and (5′- GTCCTCCTTGAAGTCGATGCCCTTCAG -3′) that generate a 336-bp product.

R26-CAG-LoxP-eNpHR3-EYFP mice were generated by crossing R26-CAG-LF-eNpHR3-EYFP and pCAG-FLPe mice^35^. R26-CAG-Frt-eNpHR3-EYFP mice were generated by crossing R26-CAG-LF-eNpHR3-EYFP and TNAP-Cre mice^36^. Removal of the floxed and/or Frt-flanked stop cassette was assessed by PCR with a forward pCAG primer (5′- AATTCCTCGACGGGGAATTCGGG -3′) and a reverse eNpHR3 primer (5′- TGAAGGGCCACGGCACTCTCGGTC -3′). PCR products of 2233 bp, 1099 bp, 1408 bp, and 274 bp were amplified from R26-CAG-LF-eNpHR3-EYFP, R26-CAG-LoxP-eNpHR3-EYFP, R26-CAG-Frt-eNpHR3-EYFP, and both stop cassette deleted R26-CAG-eNpHR3-EYFP alleles, respectively. Each line was crossed with C57BL/6 J mice several times and deposited in the RIKEN Bioresource Center (http://www.brc.riken.jp/lab/animal/en/) under the following repository stock numbers: R26-CAG-LF-eNpHR3-EYFP (RBRC05159), R26-CAG-LoxP-eNpHR3-EYFP (RBRC05160), R26-CAG-Frt-eNpHR3-EYFP (RBRC05161). Live mice, cryopreserved embryos, and sperm of the deposited knock-in strains are available from RIKEN Bioresource Center.

### Generation of R26 BAC::pCAG-LF-eNpHR3-EYFP and R26 BAC::pCAG-LoxP-eNpHR3-EYFP BAC transgenic mice

The pCAG-LF-eNpHR3-EYFP conditional expression cassette (see Fig. 1d), contains a CAG promoter, a floxed primary stop cassette containing PGK-Pgb2-Neo/Kan sequences followed by four pA sequences from bovine growth hormone pA (BGHpA), SV40pA, and rabbit beta-globin pA, an Frt-flanked stop cassette containing three pA sequences (SV40pA-TKpA-SV40pA), and eNpHR3-EYFP-WPRE-SV40pA. The Neo/Kan expression was driven by the EM7 promoter for Kan selection in *Escherichia coli*. The BAC targeting vector was generated for Rosa26 locus by cloning the 5′-upstream 807 bp and the 3′-downstream 579 bp into the pCAG-LF-eNpHR3-EYFP plasmid. For recombination, BAC targeting cassettes were excised by restriction digestion and electroporated into competent SW105 cells containing the Rosa26 BAC clone. Targeted BAC clones were selected for Kan-resistance, and correctly targeted BAC clones were identified by a panel of PCR primers and restriction digestions^37,38^. This procedure introduced the conditional eNpHR3 expression cassette into the same *XbaI* site of the Rosa26 locus that was used in the eNpHR3-knock-in mice. The modified BAC DNA was purified with a NucleoBond Xtra Midi kit (Macherey-Nagel, Düren, Germany) and injected into the pronuclei of fertilized one-cell eggs from C57BL/6J mice. About 400 fertilized eggs were injected. Potential founder mice were screened by PCR of tail DNA. Founder lines having single copy of the BAC transgene were selected by real-time PCR and used for subsequent analysis. R26 BAC::pCAG-LoxP-eNpHR3-EYFP mice were generated by crossing R26 BAC::pCAG-LF-eNpHR3-EYFP and pCAG-FLPe mice with s strategy similar to the knock-in mice. The detailed cloning strategy and complete sequence of plasmids are available on request. Below are the repository stock numbers in RIKEN Bioresource Center: R26 BAC::pCAG-LF-eNpHR3-EYFP (RBRC06583), R26 BAC::pCAG-LoxP-eNpHR3-EYFP (RBRC06584).

### Tissue preparation and immunohistochemistry

Adult mice (8–12-weeks old) were deeply anesthetized and perfused transcardially with 30 ml of PBS and 30 ml of 4% paraformaldehyde (PFA)/PBS (pH 7.2). Brains were postfixed in the perfusing solution overnight at 4 °C and then cryoprotected for 24 h in 20% sucrose in PBS. Brains were embedded in OCT compound (Sakura Finetek) and frozen at –80 °C. Cryostat sections (16- or 20-μm thickness) were incubated in 5% normal donkey serum (NDS) and 0.1% Triton X-100/PBS at room temperature (RT) for 1 h, then with primary antibodies (rabbit anti-GFP, 1:500, Invtrogen; mouse anti-NeuN, 1:400, Chemicon; rabbit anti-GABA 1:10000, Sigma; rabbit anti-Cux1, 1:100, Santa Cruz; rat anti-Ctip2, 1:500, Abcam) diluted in 0.1% Triton X-100/PBS containing 1% NDS overnight at 4 °C, washed with PBS, and then incubated with secondary antibodies conjugated to Alexa 488 or Alexa 594 (1:200, Invitrogen) for 1 h at RT. Sections were mounted with Fluormount-G (Southern Biotech.) and photographed with laser-scanning confocal microscopy (LSM510, Zeiss). Fluorescent images of sections stained with anti-GFP and anti-NeuN antibodies were acquired with a 20x objective (optical slice 2.2 μm). Fluorescent images of sections stained with anti-GFP and, anti-GABA, anti-Cux1 or anti-Ctip2 antibodies were acquired with a 63x objective (optical slice 0.7 μm).

### Brain slice preparation and *In Vitro* electrophysiological recordings

Male and female eNpHR3-EYFP-expressing mice (2–3 weeks old) were used for whole-cell recordings. 350-μm coronal brain slices, prepared as described previously^19,39,40^, were transferred to an incubation chamber filled with physiological solution containing the following in mM: 135 NaCl, 5 KCl, 1 CaCl_2_, 1 MgCl_2_, 10 HEPES, and 10 glucose, pH 7.4 with NaOH, bubbled with 100% O_2_ and incubated for at least 1 h at RT. Typically, five to seven coronal slices per brain were used for whole-cell recordings. The most anterior slice included the lateral ventricle, and the most posterior slice included the middle part of the hippocampus.

RT slices were then transferred to a recording chamber (RC-27L; Warner Instruments) on the stage of a fluorescence microscope (BX51WI; Olympus) equipped with an infrared camera (C2741-79; Hamamatsu Photonics) for infrared differential interference contrast imaging and a cooled charge-coupled device (CCD) camera (Cascade 650; Roper Scientific) for fluorescent imaging. Neurons with EYFP fluorescence were identified and subjected to *in vitro* electrophysiological recordings as described previously^19,39,40^. Electrophysiological recordings were conducted using an Axopatch 200B amplifier (Axon Instruments, Foster City, CA) by a borosilicate pipette (GC150-10, Harvard Apparatus, Holliston, MA) prepared with a micro-pipette puller (P-97, Sutter Instruments) filled with intracellular solution (4–10 MΩ), consisting of (in mM): 138 K-gluconate, 8 NaCl, 10 HEPES, 0.2 EGTA-Na_3_, 2 MgATP, 0.5 Na_2_GTP, pH 7.3 with KOH. The contents of extracellular solution were below (in mM): 124 NaCl, 3 KCl, 2 MgCl_2_, 2 CaCl_2_, 1.23 NaH_2_PO_4_, 26 NaHCO_3_ and 25 glucose, and this solution was gassed with 95% O_2_ and 5% CO_2_ in a 35 °C water bath for 30–-60 min. The osmolarity of the solutions were verified by a vapor pressure osmometer (model 5520, Wescor, Logan, UT) and found to be 280–290 and 320–330 mOsm/L for the internal and external solutions, respectively. The liquid junction potential of the patch pipette and perfused extracellular solution was estimated to be 3.9 mV and was applied to the data. These K-gluconate-based intracellular solutions were used for both current clamp and voltage clamp mode recordings. In voltage clamp recording, QX-314 (1 mM) was added in the intracellular solution. The voltage-clamp holding potential was −60 mV. Positive pressure was provided to the recording pipettes while advancing toward individual neurons in the slice. Tight seals (1.0–1.5 GΩ) were made by negative pressure. The membrane patch was then ruptured by suction. The series resistance during recording was 10–25 MΩ. The reference electrode was an Ag-AgCl pellet immersed in bath solution. During recordings, cells were superfused with extracellular solution at a rate of 1.6 mL/min using a peristaltic pump (Dynamax, Rainin, Oakland, CA).

Yellow light (563–587 nm) was generated by Light Engine SPECTRA3 (Lumencor) and illuminated through the objective lens (Olympus LUMPlan FLN 40 × 0.80 w) in the following intensities: 1, 2, 5, 10 and 20% (24 mW at 100%). We recorded responses by the five different conditions of yellow light intensity in sequence from the same cell. Recordings were made from 7 to 15 cells in each slice patch clamp analysis. We analyzed eNpHR3-EYFP-expressing neurons throughout the neocortex, including the upper and deep layers.

### Statistical analysis

Statistical analyses were performed using Prism 5.0 (Graphpad Software). Statistical differences were tested with Student’s *t* test or one-way analysis of variance (ANOVA) followed by Tukey’s post hoc test. *p* values less than 0.05 were considered to be significant.

## Supplementary information

Supplementary information.
