# Supplementary material for: Light-induced silencing of neural activity in Rosa26 knock-in and BAC transgenic mice conditionally expressing the microbial halorhodopsin eNpHR3
Source: Sci Rep. 2020 Feb 21;10:3191. doi: 10.1038/s41598-020-59984-3 (PMC7035371; doi:10.1038/s41598-020-59984-3)
Supplement: Supplementary file 1 — Supplementary information. [file 41598_2020_59984_MOESM1_ESM.pdf]

Supplementary information for:

Light-induced silencing of neural activity in Rosa26 knock-in and BAC transgenic mice conditionally expressing the microbial halorhodopsin eNpHR3

Itaru Imayoshi[1,2,3]*, Sawako Tabuchi[4], Mami Matsumoto[1,2], Satsuki Kitano[2], Hitoshi Miyachi[2], Ryoichiro Kageyama[2,3]* and Akihiro Yamanaka[4]

[1]Research Center for Dynamic Living Systems, Graduate School of Biostudies, Kyoto University, Kyoto 606-8501, Japan, [2]Institute for Frontier Life and Medical Sciences, Kyoto University, Kyoto 606-8507, Japan, [3]World Premier International Research Initiative–Institute for Integrated Cell-Material Sciences, Kyoto University, Kyoto 606-8501, Japan; [4]Research Institute of Environmental Medicine, Nagoya University, Nagoya 464-8601, Japan

*To whom correspondence should be addressed:

Itaru Imayoshi
Laboratory of Brain Development and Regeneration,
Division of Systemic Life Science,
Graduate School of Biostudies,
Kyoto University.
Shogoin-Kawahara 53, Sakyo-ku,
Kyoto 606-8507
Japan
Tel: +81-75-751-3977
Fax: +81-75-751-4807
E-mail: imayoshi.itaru.2n@kyoto-u.ac.jp

Ryoichiro Kageyama
Laboratory of Growth Regulation System,
Department of Biosystems Science,
Institute for Frontier Life and Medical Sciences, Kyoto University,
Kyoto University.
Shogoin-Kawahara 53, Sakyo-ku,
Kyoto 606-8507
Japan
Tel: 81-75-751-4011
Fax: 81-75-751-4807
E-mail: kageyama.ryoichiro.8r@kyoto-u.ac.jp

a

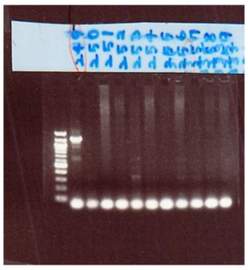

b

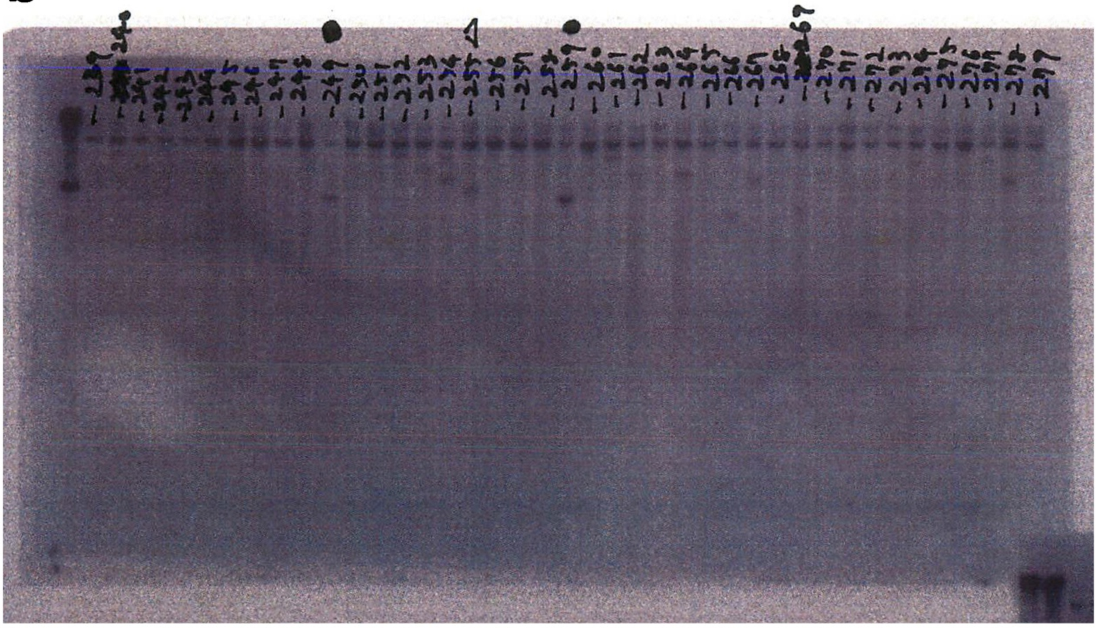

Supplementary Figure S1. The PCR data and Southern blot data, related to Figure1. The results of PCR (**a**) and Southern blot hybridization analysis (**b**) of the genomic DNA from recombinant embryonic stem cells were displayed. The results of clone-249 were highlights in figure 1b,c.
